# Pathologic Evidence of Granulomatosis With Polyangiitis as a Cause of Atrioventricular Block

**DOI:** 10.1016/j.jaccas.2025.104414

**Published:** 2025-07-30

**Authors:** Naoki Watanabe, Kenji Harada, Shigeo Kawai, Hisaya Kobayashi, Kazuomi Kario

**Affiliations:** a Division of Cardiovascular Medicine, Department of Internal Medicine, Jichi Medical University, Shimotsuke, Tochigi, Japan; b Department of Cardiology, Tochigi Medical Center Shimotsuga, Shimotsuke, Tochigi, Japan; c Department of Pathology, Tochigi Medical Center Shimotsuga, Shimotsuke, Tochigi, Japan

**Keywords:** atrioventricular block, granulomatosis with polyangiitis, pathology

## Abstract

**Background:**

Atrioventricular block (AVB) is a rare manifestation of granulomatosis with polyangiitis (GPA), and the underlying pathologic relationship between GPA and AVB remains poorly understood.

**Case Summary:**

We present the case of a 71-year-old woman who developed complete AVB and had a history of microscopic polyangiitis. Despite the absence of clinical signs indicative of GPA relapse throughout her lifetime, postmortem examination revealed microgranulomas and multinucleated giant cells within the atrioventricular node.

**Discussion:**

This case provides pathologic evidence that GPA can lead to complete heart block as an initial clinical manifestation.

**Take-Home Messages:**

GPA-associated AVB can improve with immunosuppressive therapy. GPA should be considered in the differential diagnosis of AVB of unknown etiology.

## Visual Summary

**Figure:**
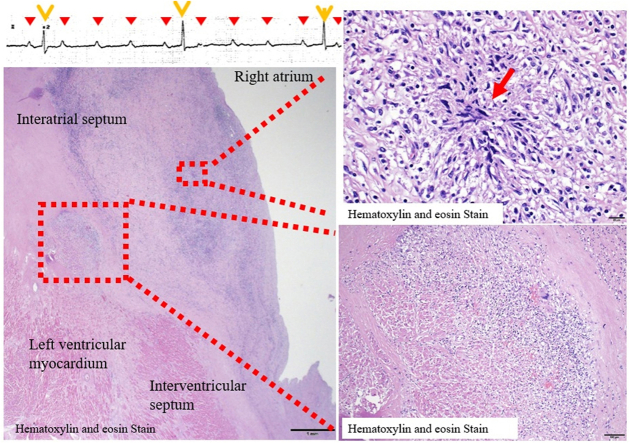

## History of Presentation

**Figure 1:**
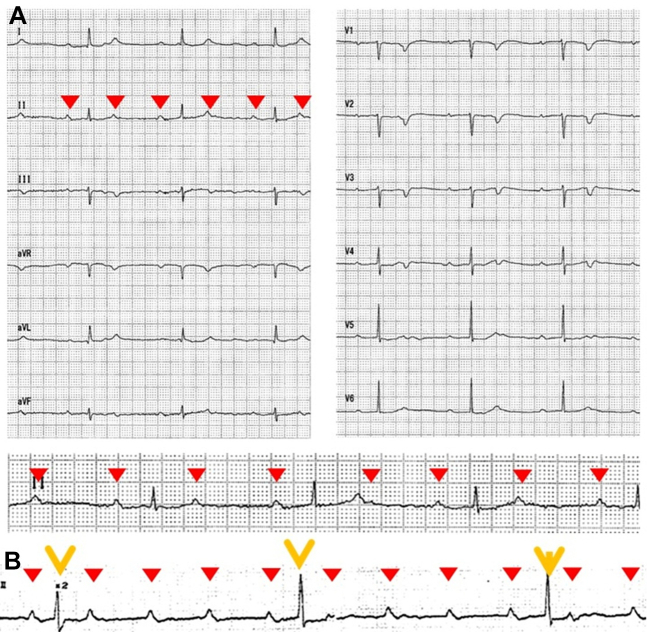
ECG Showing AVB ECG findings showing a 2:1 atrioventricular block on presentation at our hospital (A). Monitor ECG at hospitalization showing complete heart block (B). AVB = atrioventricular block; ECG = electrocardiography.

We present the case of a 71-year-old woman who was referred to our hospital after experiencing progressive dyspnea and systemic edema for 1 month. On presentation, her physical examination revealed bilateral lung crackles, elevated jugular venous pressure, and lower extremity swelling. The chest radiograph showed cardiomegaly and pulmonary edema, but no consolidations. Laboratory tests showed a serum creatinine level of 2.16 mg/dL, a brain natriuretic peptide level of 692 pg/mL, and a myeloperoxidase-antineutrophil cytoplasmic antibody (MPO-ANCA) level of approximately 0.5 U/mL. Electrocardiography revealed a 2:1 atrioventricular block (AVB) (Figure 1A). Transthoracic echocardiography demonstrated well-preserved left ventricular systolic function, mild enlargement of cardiac cavities, mild mitral regurgitation, and mild tricuspid regurgitation with pulmonary hypertension (estimated pulmonary artery systolic pressure of 46.3 mm Hg).

**Take-Home Messages**

• This case provides pathologic evidence that GPA can present as complete heart block.
• Given that GPA-associated atrioventricular block may improve with immunosuppressive therapy, GPA should be considered in the differential diagnosis of atrioventricular block of unknown etiology.

## Past Medical History

Eight years before presentation at our hospital, at age 63, she was clinically diagnosed with microscopic polyangiitis (MPA) based on rapidly progressive glomerulonephritis, mononeuropathy multiplex, positive MPO-ANCA, and negative proteinase 3–ANCA; no renal biopsy was conducted at that time. Despite impaired renal function (serum creatinine 2.30 mg/dL), her MPA was well controlled over the next 8 years with daily immunosuppressive therapy consisting of 7 mg of prednisolone and 100 mg of mizoribine. Five years ago, the patient was diagnosed with idiopathic femoral head necrosis secondary to steroid use. Although surgical intervention was indicated, conservative management was chosen because of the high perioperative risk associated with MPA. Consequently, she was unable to walk and used a wheelchair.

## Differential Diagnosis

AVB can result from a variety of underlying conditions, including electrolyte imbalances, myocardial ischemia, and cardiomyopathies. Among these, cardiac sarcoidosis is a well-established cause of AVB. Therefore, clinicians should be vigilant in evaluating for signs suggestive of these etiologies, such as elevated serum soluble interleukin-2 receptor levels, elevated serum angiotensin-converting enzyme levels, or thinning of the basal ventricular septum on imaging studies. However, it is important to recognize that the etiology of AVB remains idiopathic in many elderly patients.

## Investigations

There was no evidence of electrolyte disturbances, ischemic heart disease, or cardiomyopathy (Table 1). Notably, there were no signs of cardiac sarcoidosis, such as elevated serum soluble interleukin-2 receptor levels, elevated serum angiotensin-converting enzyme levels, or thinning of the basal ventricular septum on echocardiography. Therefore, a diagnosis of idiopathic complete AVB was made.

**Table 1:** Laboratory Examinations on Admission

Test	Value
Peripheral blood tests	
WBC	11,500/mL
Neutrophil	94.3%
Eosinophil	0.1%
Lymphocyte	3.6%
Monocyte	1.7%
Basophil	0.3%
Hb	9.1 g/dL
PLT	37.9 × 10^4^/mL
Immunological tests	
BNP	692 pg/mL
MPO-ANCA	0.3 U/mL
IgG	1,111 mg/dL
ACE	15.3 U/L
sIL-2R	526 U/mL
Biochemistry tests	
TP	6.5 g/dL
Albumin	3.1 g/dL
T-bill	0.7 mg/dL
BUN	52 mg/dL
Creatinine	2.16 mg/dL
eGFR	18.2 mL/min/1.73 m^2^
AST	10 U/L
ALT	10 U/L
LDH	222 U/L
Na	136 mEq/L
K	4.6 mEq/L
Cl	103 mEq/L
CRP	3.72 mg/dL
TSH	0.798 μU/mL
F-T4	1.3 ng/dL

ACE = angiotensin converting enzyme; ALT = alanine aminotransferase; AST = aspartate aminotransferase; BNP = brain natriuretic peptide; BUN = blood urea nitrogen; Cl = chloride; CRP = C-reactive protein; F-T4 = free thyroxine 4; eGFR = estimated glomerular filtration rate; Hb = hemoglobin; IgG = immunoglobulin G; K = potassium; LDH = lactate dehydrogenase; MPO-ANCA = myeloperoxidase-antineutrophil cytoplasmic antibodies; Na = sodium; PLT = platelets; sIL-2R = soluble interleukin-2 receptor; T-bil = total bilirubin; TP = total protein; TSH = thyroid stimulating hormone; WBC = white blood cells.

## Management

Heart failure secondary to AVB was diagnosed, and treatment of primarily diuretics was initiated. Her symptoms gradually improved with the heart failure treatment. However, despite being asymptomatic, a transient complete AVB was detected on her continuous electrocardiography monitoring (Figure 1B). Permanent pacemaker implantation (PMI) was planned due to idiopathic complete AVB causing heart failure. However, she developed pyelonephritis, leading to a postponement of the procedure until after antimicrobial treatment. The permanent PMI was further delayed because of a series of infections, including herpes zoster at the planned PMI site and acute cholangitis. In addition, she developed sepsis for which the etiology could not be undetermined. Unfortunately, despite antibiotic treatment, she died of multiorgan failure due to sepsis on the 89th day of hospitalization.

## Outcome and Follow-Up

**Figure 2:**
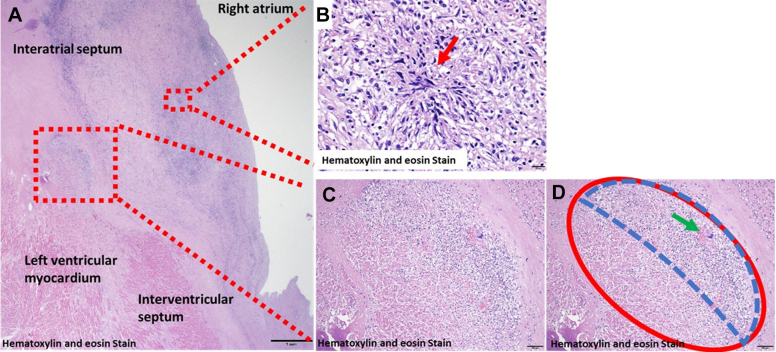
Postmortem Pathologic Findings of GPA Causing AVB Microgranulomas were observed extending from the right atrial septum to the right fibrous trigone (A, B, red arrow). The compact node of the atrioventricular node (D, red area) and His bundles contained multinucleated giant cells (D, green arrow) and marked chronic inflammatory cell infiltration (D, blue area). The findings proved that GPA caused the AVB. AVB = atrioventricular block; GPA = granulomatosis with polyangiitis.

**Figure 3:**
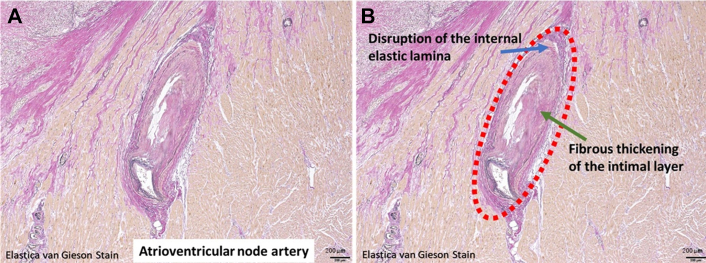
Postmortem Pathologic Findings of Myocardial Vasculitis Postmortem pathologic examination of the myocardial specimen showing disruption of the internal elastic lamina (B, blue arrow) and fibrous thickening of the intimal layer (B, green arrow) of the atrioventricular node artery, which are indicative of healed vasculitis.

Necropsy revealed lymphocytic, histiocytic, and eosinophilic infiltration in the myocardial interstitium of the left ventricle, consistent with myocarditis (Figures 2A and 2B). Disruption of the internal elastic lamina and fibrous thickening of the intimal layer of the atrioventricular node artery indicated healed vasculitis (Figure 3). Microgranulomas were observed extending from the right atrial septum to the right fibrous trigone. Notably, multinucleated giant cells and marked chronic inflammatory cell infiltration were found in the compact node of the atrioventricular node and His bundle, confirming that granulomatosis with polyangiitis (GPA) was responsible for the AVB (Figures 2A to 2D). The left lung exhibited subacute alveolar hemorrhage with vascular wall disruption, and alveolar hemorrhage was noted at the site of infiltrative consolidation on computed tomography conducted immediately before death.

## Discussion

In the present case, the patient was clinically diagnosed with MPA based on the overall clinical presentation and serologic findings. However, postmortem examination revealed that the definitive diagnosis was GPA, which was identified as the underlying cause of the AVB. MPO-ANCA is positive in approximately 50% of patients with MPA, whereas negative proteinase 3–ANCA is positive in approximately 65% of patients with GPA. Notably, it has been reported that up to 20% of patients with GPA may also test positive for MPO-ANCA.^1^ In the United Kingdom, patients with MPO-ANCA–positive vasculitis account for 30% of patients with ANCA-associated vasculitis, whereas in Japan, this figure is 84%.^2^ Furthermore, of 33 patients with newly diagnosed GPA from April 2009 to December 2010, in 22 Japanese tertiary care institutions, 15 patients were positive for MPO-ANCA alone.^3^ Given these findings from the literature, clinicians should exercise particular caution when diagnosing MPA based solely on MPO-ANCA positivity and clinical features, especially in Japanese patients, as illustrated in the present case.

Cardiac involvement is a relatively well-recognized manifestation of GPA among patients with ANCA-associated vasculitis,^4^ and the presence of AVB further supports the final diagnosis of GPA in this case. However, cardiac involvement remains a rare occurrence overall.^4^ In a 2015 cohort study of 517 patients with GPA, 17 (3.3%) had cardiac involvement. Among these 17 patients, pericarditis was the most common form of cardiac involvement, and there was only 1 case of AVB.^5^ Although 18 cases of AVB in GPA have been reported, none of these included pathologic evidence.^6^, ^7^, ^8^, ^9^ To our knowledge, there has been only 1 reported necropsy case with granuloma in the atrioventricular node. However, that case did not provide evidence of the AVB.^10^

In our present case, necropsy revealed GPA relapse involving the heart and the lung. At the time of our earlier diagnosis of transient complete AVB, the chest computed tomography showed no evidence of infiltrating consolidation. Therefore, the complete heart block was the first involvement of GPA relapse in this case. Furthermore, based on the available literature, this is the first reported case providing pathologic evidence that GPA can directly cause AVB.

In addition, of the 18 previously reported cases of GPA complicated by AVB, 6 showed improvement of conduction disturbances with immunosuppressive therapy,^4^, ^5^, ^6^, ^7^, ^8^, ^9^ underscoring the importance of timely diagnosis. Moreover, diagnosis of GPA as a cause of AVB may help avoid unnecessary pacemaker implantation. Our present findings thus suggest that other involvements of GPA should be considered in all patients with complete heart block. Especially in patients with ANCA-associated vasculitis and AVB, we must check for signs of GPA relapse.

Recognizing GPA as the underlying cause of AVB may help prevent unnecessary pacemaker placements. Therefore, when diagnosing complete heart block, it is essential to evaluate for other signs of GPA, particularly in patients with ANCA-associated vasculitis.

## Conclusions

We experienced a case of GPA presenting with complete heart block. Although GPA is rarely associated with AVB, the pathology examination suggests that it can be a first clinical finding. Furthermore, GPA-associated AVB can be improved by immunosuppressive therapy. GPA should therefore be considered for AVB of unknown cause.

**Visual Summary:**
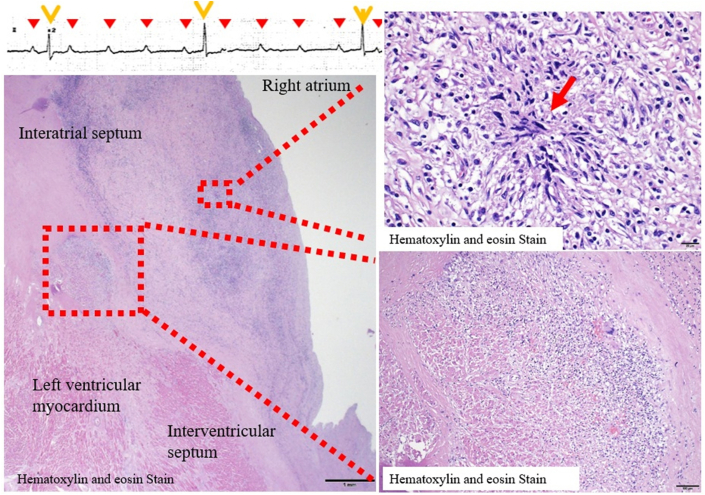
Pathological Evidence that GPA can Cause Complete Heart Block

## Consent

The authors confirm that written consent for the submission and publication of this case report was obtained from the patient's family, after the patient's death, in line with COPE guidance.

## Funding Support and Author Disclosures

The authors have reported that they have no relationships relevant to the contents of this paper to disclose.
